# Spreading depolarization remarkably exacerbates ischemia-induced tissue acidosis in the young and aged rat brain

**DOI:** 10.1038/s41598-017-01284-4

**Published:** 2017-04-25

**Authors:** Ákos Menyhárt, Dániel Zölei-Szénási, Tamás Puskás, Péter Makra, M. Tóth Orsolya, Borbála É. Szepes, Réka Tóth, Orsolya Ivánkovits-Kiss, Tihomir P. Obrenovitch, Ferenc Bari, Eszter Farkas

**Affiliations:** Department of Medical Physics and Informatics, Faculty of Medicine & Faculty of Science and Informatics, University of Szeged, H-6720 Szeged, Korányi fasor 9, Hungary

## Abstract

Spreading depolarizations (SDs) occur spontaneously in the cerebral cortex of subarachnoid hemorrhage, stroke or traumatic brain injury patients. Accumulating evidence prove that SDs exacerbate focal ischemic injury by converting zones of the viable but non-functional ischemic penumbra to the core region beyond rescue. Yet the SD-related mechanisms to mediate neurodegeneration remain poorly understood. Here we show in the cerebral cortex of isoflurane-anesthetized, young and old laboratory rats, that SDs propagating under ischemic penumbra-like conditions decrease intra and- extracellular tissue pH transiently to levels, which have been recognized to cause tissue damage. Further, tissue pH after the passage of each spontaneous SD event remains acidic for over 10 minutes. Finally, the recovery from SD-related tissue acidosis is hampered further by age. We propose that accumulating acid load is an effective mechanism for SD to cause delayed cell death in the ischemic nervous tissue, particularly in the aged brain.

## Introduction

Recurrent spreading depolarizations (SDs) have been recognized to be central to the progression of cerebral tissue damage following subarachnoid hemorrhage, stroke or traumatic brain injury^1^. Moreover, the association between SD occurrence after an acute brain insult and poor neurological outcome has led to the promotion of SD as a causal biomarker to be assessed in neurocritical care, to indicate the degree of metabolic failure in the nervous tissue^2^. Finally, the inhibition of SD occurrence in the injured brain of patients has been put forward as a therapeutic strategy to limit the expansion of primary lesions caused by hemorrhage or trauma^3^. Nevertheless, specific mediators of SD-related injury are not clear and should be explored in order to focus efforts to inhibit the generation of injurious SDs.

The negative shift of the direct current (DC) potential is the hallmark of SD. In addition to its prominent impact on brain ion homeostasis^4^, SD induces fundamental metabolic changes in the tissue, reflected, for example, by the rapid consumption of glucose^5, 6^, increased oxygen utilization^7, 8^, reduction of tissue ATP content^6^, accumulation of lactate^5, 9^, and reduction of tissue pH^10^. In the ischemic cortex, the SD-created metabolic demand to re-establish resting membrane potential of neurons exceeds effective nutrient and oxygen supply provided by the insufficient cerebral circulation, thereby worsening neuronal damage^11, 12^. The most serious consequence of the occurrence of recurrent SDs after focal ischemic stroke is the conversion of viable but non-functional penumbra tissue into the irrevocably damaged core region, ultimately increasing lesions size and worsening neurological outcome^1^.

A relevant, sensitive indicator of cellular metabolism is tissue pH, which is substantiated by the rapid occurrence of an acidic shift with neuronal activity^13^. Furthermore, excessive acidosis in the brain does not only designate altered metabolism, but has long been considered to cause neuronal injury possibly by generating free radicals, disrupting intracellular signal transduction pathways, and inducing DNA fragmentation^14^. Extracellular pH changes with SD contain a transient shift from 7.35 to 6.95 in the intact rodent cortex^10^. This relatively mild, brief acidosis by itself will not harm neurons, but could be crucial for neuronal loss or survival when repeatedly superimposed (i.e. recurrent SDs) on ischemia-induced acidosis characterized by pH values around 6.9 in the penumbra^15^. It is, therefore, reasonable to propose that there is indeed such an additive acid load, which would be an indicator of or, more importantly, a contributor to the SD-related metabolic crisis and related neurodegeneration in ischemic tissue^16^. Direct measurement of tissue pH in the ischemic cortex has not been conducted to confirm or discard the validity of this concept, yet. Still, supportive experimental data have shown that hypoxia in combination with mild acidosis increases the duration of SD-related acidosis in brain slices *in vitro* ^17^, and lactate accumulation with SD is greater in the ischemic penumbra with respect to SDs propagating across the intact cortex^18^.

Here we created penumbra-like conditions by inducing global forebrain ischemia in the rat, and investigated SD-related pH transients to prove that the occurrence of SD in the ischemic cortex is building up acid load to a level which has been recognized to cause tissue damage^19^. Since the aged brain is more susceptible to SD-related injury^20–22^, we hypothesized that SD-related acidosis must also be graver in the old brain. Therefore we conducted our experiments in old, as well as in young animals. This approach fortifies the translational potential of this research, because stroke occurs more frequently^23^, and the core region grows more rapidly at the expense of the penumbra at older age^24^.

To achieve the goals set, we relied on conventional pH-sensitive microelectrodes, and introduced tissue pH imaging. Even though pH-sensitive microelectrodes provide excellent extracellular pH (pHe) signal, additional, spatial resolution is also required to follow SDs that occur spontaneously at unpredictable sites in the ischemic cortex, and propagate across regions of various metabolic status. Neutral Red (NR) is a vital dye that indicates changes of intracellular pH (pHi) in the brain^25^, and has been applied successfully to follow spreading acidification and depression in the cerebellum^26, 27^. We adapted NR imaging to monitor pHi changes with SD in the rat cerebral cortex, and combined it with laser speckle contrast analysis (LASCA)-based cerebral blood flow (CBF) imaging to directly relate tissue pH changes to CBF variations.

## Results

An overview of the physiological variables is presented in Table 1.

**Table 1:** Obtained values and statistical evaluation of arterial blood pH, and the level of metabolites relevant for pH adjustment in arterial blood, and mean arterial blood pressure (MABP).

Variable	Phase of experiment	Age		Multivariate analysis (factors)			
		Young (n = 16)	Old (n = 19)	Stat. values	Ischemia	Age	Ischemia x Age
pH	Baseline	7.37 ± 0.03	7.40 ± 0.02	F	0	48.05	11.85
	Ischemia	7.34 ± 0.05	7.43 ± 0.04	p	0.983	0.0001**	0.001**
pCO_2_	Baseline	34.66 ± 5.4	34.69 ± 5.1	F	4.204	4.435	4.506
	Ischemia	41.34 ± 8.2	34.57 ± 7.6	p	0.044*	0.039*	0.038*
HCO_3_^−^	Baseline	20.13 ± 3.1	21.61 ± 3.0	F	5.846	2.632	0.073
	Ischemia	22.23 ± 3.2	23.28 ± 3.7	p	0.018 *	0.109	0.788
Lactate (mmol/l)	Baseline	0.80 ± 0.3	0.71 ± 0.2	F	1.247	5.113	0.499
	Ischemia	0.90 ± 0.3	0.74 ± 0.2	p	0.268	0.027*	0.482
MABP	Baseline	98.6 ± 10.0	89.1 ± 12.8	F	36.583	0.583	4.376
	Ischemia	101.8 ± 10.9	102.2 ± 13.1	p	0.0001	0.450	0.044*
	Reperfusion	96.0 ± 9.7	96.8 ± 13.2				

Data from both the Electrophysiology and Imaging series of experiments are shown and are given as mean±stdev. Level of significance is given as *p < 0.05 and **p < 0.01.

SD occurrence was confirmed by the transient negative shift of the DC potential in all experiments; comprehensive quantitative analysis of the DC potential shift was conducted in the electrophysiology series. In accordance with previous data^22^, the amplitude of the negative DC potential shift was conserved over the distinct phases of the experiments and age groups (two-way ANOVA: F_phase_ = 0.773, F_age_ = 0.460). The duration of the negative DC potential shift, was sensitive to the variables, as expected (two-way ANOVA: F_phase_ = 23.107, F_age_ = 9.243). In particular, ischemia considerably elongated SD duration (e.g. young: 89.9 ± 46.6 vs. 27.4 ± 11.3 s, ischemia vs. baseline).

### Tissue pH imaging using Neutral Red with reference to pH-sensitive microelectrodes

**Figure 1:**
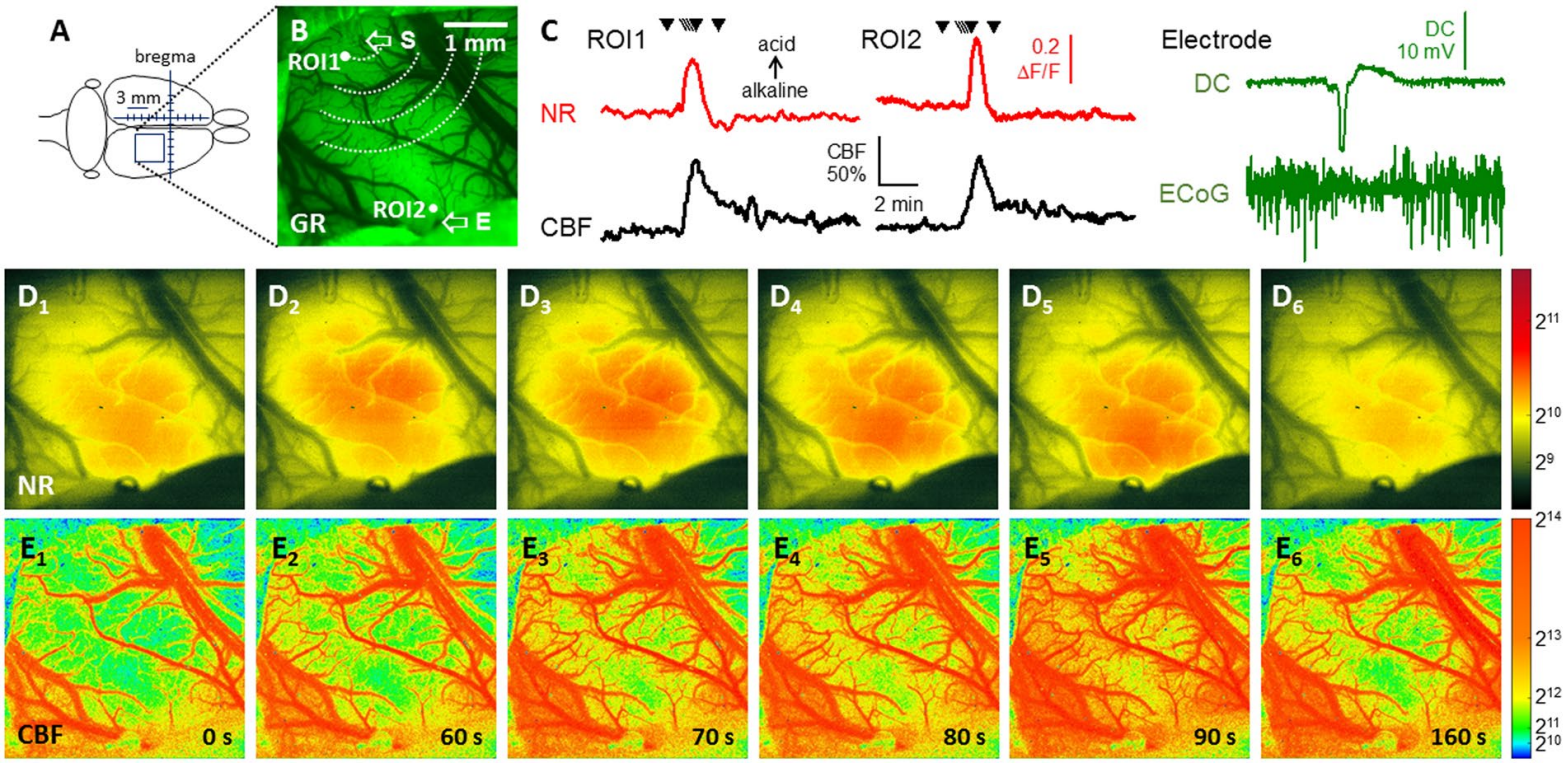
Representative raw image sequences and graphs illustrate synchronous changes in Neutral Red (NR) fluorescence, cerebral blood flow (CBF), direct current (DC) potential and the electrocorticogram (ECoG) with the propagation of a spreading depolarization (SD) wave in the intact cerebral cortex of a young rat. (**A**) Position of the closed cranial window over the parietal cortex. (**B**) The cortical surface at green light illumination. Two regions of interest (ROI) indicate the origin of NR and CBF traces shown in Panel C. The tip of the capillary used to trigger SD (S), and the penetration site of the glass capillary electrode (E) inserted to acquire electrophysiological signals are shown with arrows. (**C**) Traces demonstrate SD-related relative changes in NR fluorescence (red) and CBF (black). The occurrence of SD was confirmed by the negative shift of the DC potential and the transient depression of the ECoG (green). Small black triangles pointing down over the NR traces designate the origin of the representative images shown in Panels D,E. (**D**) Pseudo-colored images of NR fluorescence demonstrate a propagating increase of fluorescence intensity associated with SD; the original images relying on a 16-bit grayscale. A scale bar to the right represents the used color range. (**E**), Pseudo-colored perfusion maps based on laser speckle contrast images (in 16-bit resolution) show the CBF response with SD, and display speckle contrast perfusion units. Warmer colors represent higher CBF. A scale bar to the right represents the used ranges of gray levels.

In all experiments, each SD was accompanied by a highly reproducible, transient elevation of NR fluorescence intensity (Fig. 1C, red traces), which propagated across the field of view discernably from the site of SD elicitation in a radial fashion (Fig. 1D). The captured pH transients were undoubtedly related to SDs as confirmed by the typical negative DC shift and the simultaneous depression of the electrocorticogram (ECoG) recorded by an intra-cortical microelectrode (Fig. 1C, green trace). Furthermore, the propagation of the NR fluorescence intensity elevation was spatially and temporally locked with the SD-coupled CBF response visualized by LASCA (Fig. 1E).

**Figure 2:**
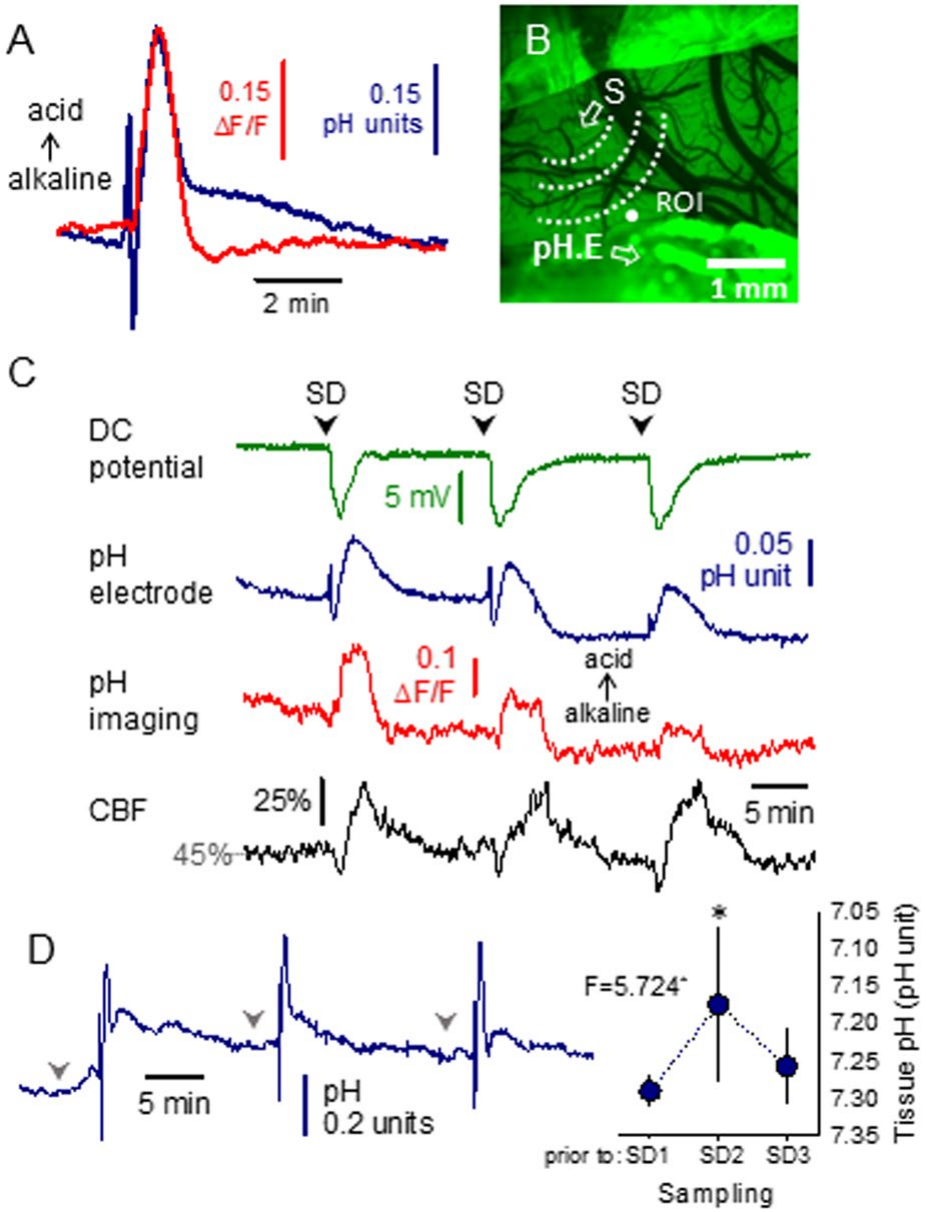
Changes in Neutral Red (NR) fluorescence indicate tissue pH variations with spreading depolarization (SD) and correlate with the signature of SD acquired with pH-sensitive microelectrodes. (**A**) Overlay of traces recorded with pH-sensitive microelectrodes (blue) and imaging of NR fluorescence (red), taken during the propagation of recurrent SD waves in the intact cortex of young rats. Note that acidosis (decreasing pH) is depicted upward. Each trace is the mean of individual events (SD2) obtained from separate experiments (n = 6). (**B**) A field of view revealed by the cranial window at green light illumination. A region of interest (ROI) indicates the origin of NR and cerebral blood flow (CBF) traces shown in panel C. Note the tip of the capillary used to trigger SD (S), and the position of the pH sensitive and reference electrodes (pH.E) inserted into the cortex to acquire local changes in tissue pH and direct current (DC) potential. (**C**) Synchronous traces obtained during ischemia from the cranial window of a young rat shown in panel B. Note the good correspondence of pH signals acquired with a pH-sensitive microelectrode (blue) and NR imaging (red). The DC potential trace (green) confirms SD occurrence. (**D**) A representative trace of tissue pH variations acquired with a pH-sensitive microelectrode from the intact cortex of a young rat indicates that baseline tissue pH remains elevated after the first SD event, and returns to pre-SD1 level gradually. Mean values of tissue pH at the time points indicated by gray arrow heads above the representative trace are shown in the plot to the right. Data are given as mean ± stdev. Statistical analysis relied on a repeated measures ANOVA paradigm (F value is given in the plot, level of significance was determined as *p < 0.05) followed by pairwise comparisons (*p < 0.05 vs. prior to SD1).

The pH-sensitive microelectrodes applied in our study revealed three subsequent phases of pHe variations associated with SD: (i) a brief, initial acidic shift immediately followed by (ii) a rapid, short alkaline shift, and (iii) a final, longer-lasting, dominant, transient acidosis (Fig. 2A, blue trace). Tissue pH remained elevated in the intact cortex especially after SD1, and original baseline was achieved gradually, by the time SD3 was elicited (Fig. 2D).

The kinetics of the increase of NR fluorescence intensity with each SD was comparable to that of the dominant tissue acidosis acquired with pH-sensitive microelectrodes (Fig. 2). In particular, the duration of this marked acidosis measured at half amplitude was nearly identical as assessed with the two distinct methods (for baseline SDs in young rats, pH-sensitive microelectrode: 40.2 ± 8.1 s, NR imaging: 39.8 ± 13.8 s). However, the initial, brief acidic and subsequent alkaline shifts obvious on the pH-sensitive microelectrode signal did not appear consistently on the traces obtained with NR imaging (Fig. 2A). Finally, the recovery from SD-related acidosis as acquired by microelectrodes contained an additional, slow component responsible for a more gradual recovery of pHe, which did not occur on the pHi signature of SD provided by NR imaging (Fig. 2). A clear limitation of NR imaging concerned the late phase of the experiments. During reperfusion, NR imaging failed to expose SD-related pHi changes reliably (the amplitude of NR fluorescence intensity elevation was very small, if any), despite of the consistent DC signature of and CBF response to SDs. In contrast, pH-sensitive microelectrodes provided good quality pHe signals with SDs during the phase of reperfusion, similar to SD-related pH transients recorded in the intact cortex under baseline conditions.

### Enhanced tissue acidosis associated with spreading depolarization in the ischemic cortex

**Figure 3:**
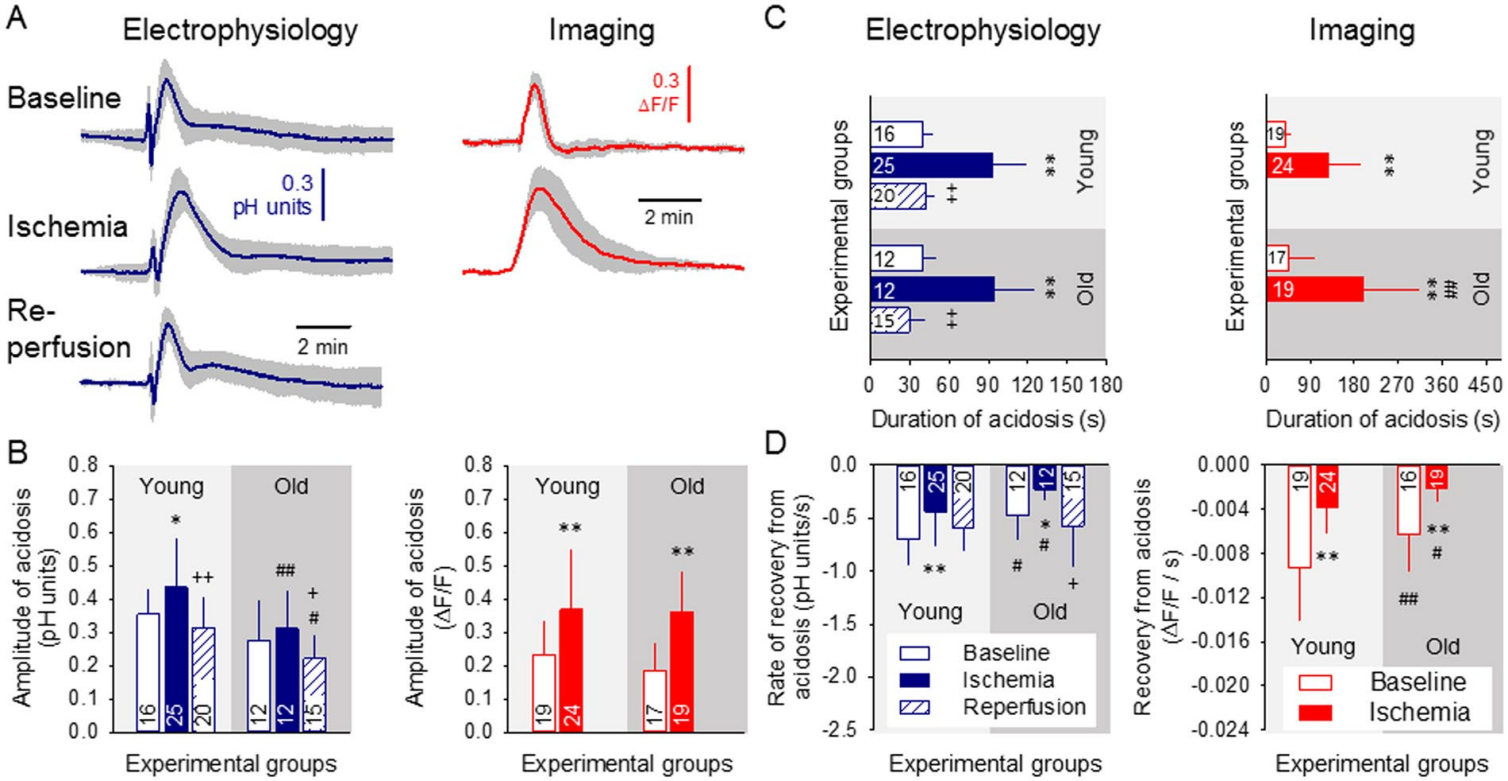
Tissue acidosis with spreading depolarization (SD) is augmented during ischemia, and recovers slower in the aged brain. In each panel, data obtained with pH-sensitive microelectrodes are shown on the left, and data derived from Neutral Red fluorescence imaging are shown on the right. (**A**) Tissue pH variations associated with SDs occurring during the three subsequent phases (i.e. baseline, ischemia, reperfusion) of the experiments. Each trace is the average of individual ones taken from separate animals, and are presented as mean ± stdev (n = 6/8). (**B**) Relative amplitude of acidosis. (**C**) Duration of acidosis at half amplitude. (**D**) Rate of recovery from acidosis (i.e. slope). Data are given as mean ± stdev; sample size (number of events) is shown in each bar. For the evaluation of statistical significance, a two-way analysis of variance (ANOVA) paradigm considering age and the phase of the experiment as its factors was applied. Level of significance for the ANOVA was defined as *p < 0.05. Significant differences between groups were determined by a Fisher post hoc test as follows: *p < 0.05 and **p < 0.01, vs. respective Baseline; ^+^p < 0.05 and ^++^p < 0.01, vs. respective Ischemia; ^#^p < 0.05 and ^##^p < 0.01, vs. respective Young.

Under ischemia, acidosis associated with evoked SDs was substantially augmented (Fig. 3A). This was reflected by the greater relative amplitude with respect to baseline SDs (for young animals, microelectrode: 0.43 ± 0.15 vs. 0.36 ± 0.07 pH units; NR imaging: 0.37 ± 0.18 vs. 0.23 ± 0.10 ΔF/F) (Fig. 3B); the longer duration (for young animals, microelectrode: 93.1 ± 26.3 vs. 40.2 ± 8.1 s; NR imaging: 127.5 ± 64.2 vs. 39.8 ± 13.8 s) (Fig. 3C), the greater magnitude expressed as area under the curve (for young animals, microelectrode: 2415 ± 869 vs. 855 ± 322 pH unit x s; NR imaging: 57.4 ± 50.9 vs. 9.8 ± 5.9 ΔF/F x s), and the slower recovery from acidosis (for young animals, microelectrode: 0.44 ± 0.33 vs. 0.70 ± 0.23 pH units/s; NR imaging: 0.004 ± 0.002 vs. 0.009 ± 0.005 ΔF/F/s) (Fig. 3D). During reperfusion, the quantitative measures of SD-related acidosis returned to near pre-ischemic values (e.g. for young animals, relative amplitude 0.31 ± 0.09 vs. 0.36 ± 0.07 pH units; duration: 42.0 ± 8.0 vs. 40.2 ± 8.1 s) (Fig. 3B,C).

Aging also had noticeable impact on the pH transients with SDs. The recovery from acidosis was significantly slower in the old group as compared with the young during baseline (electrophysiology: 0.47 ± 0.24 vs. 0.70 ± 0.26 pH units/s; NR imaging: 0.006 ± 0.003 vs. 0.009 ± 0.005 ΔF/F/s) and ischemia (electrophysiology: 0.23 ± 0.12 vs. 0.44 ± 0.005 pH units/s; NR imaging: 0.002 ± 0.001 vs. 0.004 ± 0.002 ΔF/F/s) (Fig. 3D). Further, NR imaging showed longer-lasting acidosis during ischemia in the old group with respect to the young (197.2 ± 116.7 vs. 127.5 ± 64.2 s).

### Cerebral blood flow response to spreading depolarization

**Figure 4:**
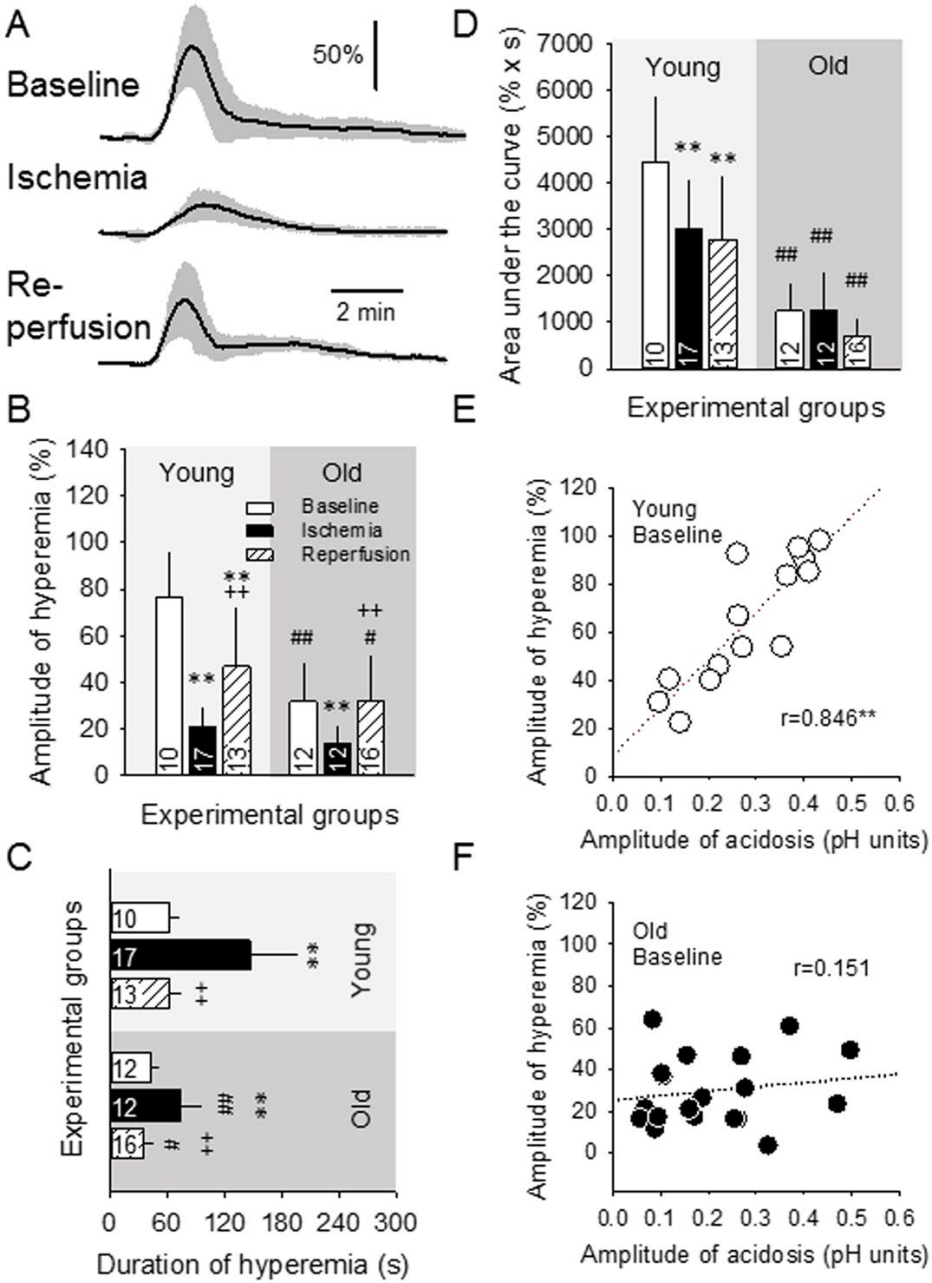
The impact of age and the association of tissue pH with the cerebral blood flow (CBF) response to spreading depolarization (SD). All data were obtained with laser Doppler flowmetry and pH sensitive microelectrodes (the latter applicable for panels E and F). (**A**) CBF variations associated with SDs, occurring in subsequent phases (i.e. baseline, ischemia, reperfusion) of the experiments in young rats. Each trace is the average of individual ones taken from separate experiments, and are presented as mean ± stdev (n = 6/8). (**B**) Relative amplitude of hyperemia. (**C**) Duration of hyperemia at half amplitude. (**D**) Magnitude of hyperemia expressed as area under the curve. (**E**) The relative amplitude of SD-related tissue acidosis and hyperemia show a direct, linear, positive correlation in the intact cortex of young rats. (**F**) The above correlation is lost in the intact cortex of old animals. Data in the bar charts are given as mean ± stdev, sample size (number of events) is shown in each bar. For the evaluation of statistical significance, a two-way analysis of variance (ANOVA) paradigm considering age and the phase of the experiment as its factors was applied. Level of significance for the ANOVA was defined as *p < 0.05 and **p < 0.01. Significant differences between groups were determined by a Fisher post hoc test as follows: *p < 0.05 and **p < 0.01, vs. respective Baseline; ^+^p < 0.05 and ^++^p < 0.01, vs. respective Ischemia; ^#^p < 0.05 and ^##^p < 0.01, vs. respective Young. Normality of data in E and F was tested by the Shapiro-Wilk test (Young acidosis p = 0.293, Young hyperemia p = 0.155, Old acidosis p = 0.051, Old hyperemia p = 0.151), followed by Spearman correlation analysis (**p < 0.01).

The CBF response to evoked SDs was predominantly hyperemic in all experimental groups, except for a few hypoemic transients observed under ischemia. The onset of hyperemia was delayed with respect to the onset of acidosis (24.5 ± 23.5 s). The relative amplitude of hyperemia markedly decreased (for young: 20.8 ± 8.9 vs. 76.3 ± 119.6%), while its duration more than doubled (for young: 146.4 ± 49.7 vs. 61.4 ± 11.6 s), under ischemia with respect to baseline (Fig. 4). As a consequence, the magnitude of hyperemia expressed as area under the curve was significantly smaller during ischemia (3009 ± 1073 vs. 4428 ± 1436% x s) (Fig. 4D). The amplitude and magnitude of the hyperemic response during reperfusion was not re-established to pre-ischemic values, but remained considerably smaller, the magnitude being even similar to that under ischemia (for young: 46.9 ± 25.3 vs. 76.3 ± 19.6%; 2788 ± 1362 vs. 4428 ± 1436% x s) (Fig. 4). The impact of age was most of all reflected by the prominent decrease of the magnitude of hyperemia under all three phases of the experiments (i.e. baseline, ischemia and reperfusion) (Fig. 4D).

We examined whether the evolution of hyperemia was associated with tissue pH changes during baseline and found in the young animals that the higher amplitude of acidosis clearly coincided with the higher amplitude of hyperemia (r = 0.846**) (Fig. 4E). In contrast, such correlation could not be discovered in the old animals (r = 0.151) (Fig. 4F). The duration of hyperemia appeared to be unrelated to the duration of acidosis in both age groups (e.g. young baseline: r = 0.371; old baseline: r = 0.193).

### Aggravation of ischemia-induced tissue acidosis by spontaneous spreading depolarization

**Figure 5:**
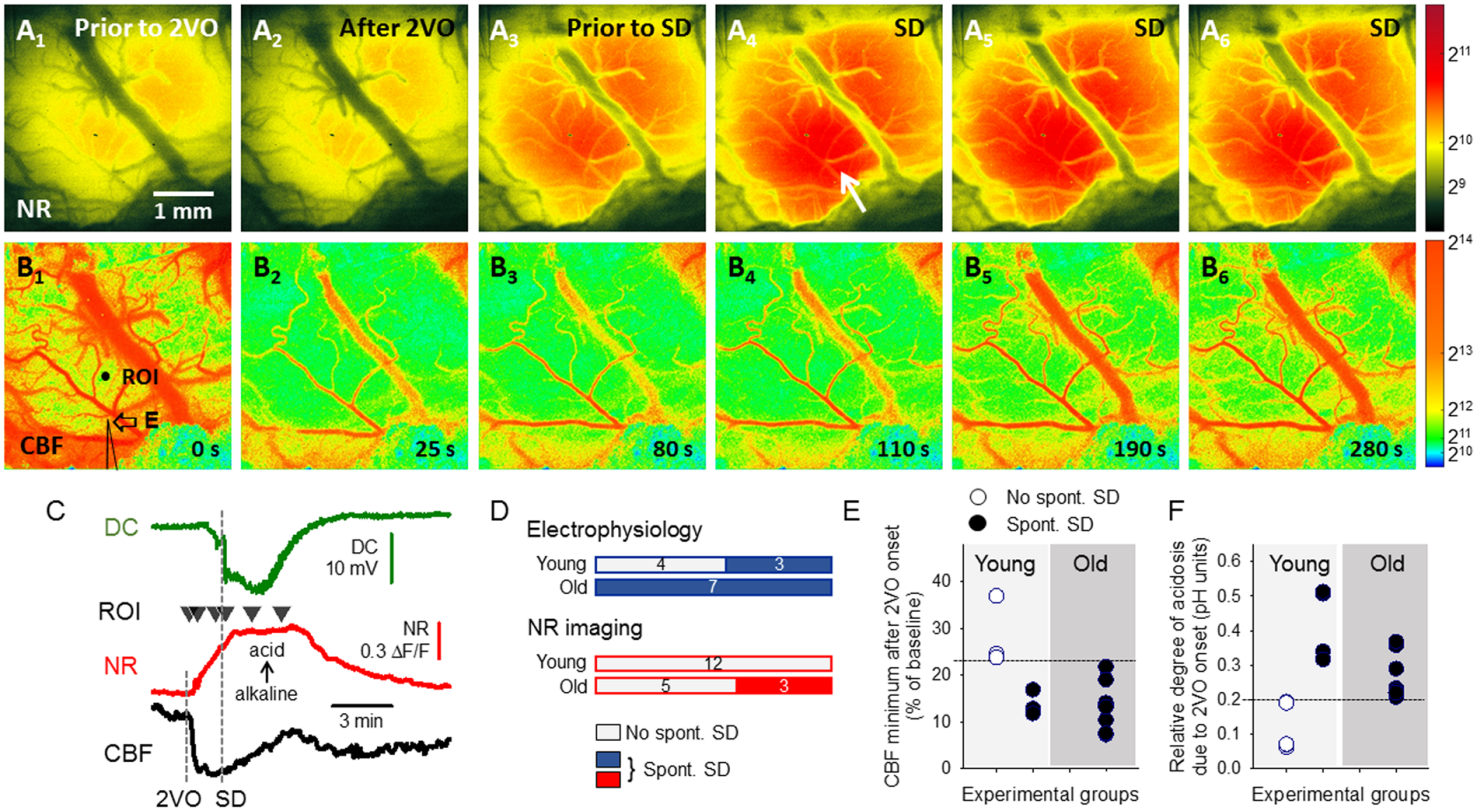
Representative raw image sequences and graphs displaying relative variations illustrate synchronous changes in Neutral Red (NR) fluorescence (**A**), cerebral blood flow (CBF) (**B**), and direct current (DC) potential (**C**), with the propagation of a spontaneous spreading depolarization (SD) wave in the ischemic cerebral cortex of an old rat. Pseudo-colored NR images in Panel A were derived from monochrome images relying on a 16-bit grayscale, while pseudo-colored CBF maps (16-bit resolution) in Panel B display speckle contrast perfusion units. Scale bars to the right represent the used ranges of gray levels. In Panel A4, the entry to the field of view and direction of propagation of a spontaneous SD is shown by a white arrow. In Panel B1, the penetration site of the glass capillary electrode (**E**) inserted to acquire electrophysiological signals is shown with an arrow, and a region of interest (ROI) indicates the origin of NR and CBF traces shown in Panel C. (**C**) Traces demonstrate the onset of ischemia (2VO) and the evolution of a spontaneous SD as shown by the DC potential signature (green), and the ischemia or SD-related relative changes in NR fluorescence (red) and CBF (black). Small black triangles pointing down over the NR traces designate the origin of the representative images shown in Panels A,B. (**D–F**) Conditions that favor the spontaneous occurrence of SD shortly after ischemia onset are presented. (**D**) Ratio of experiments devoid of spontaneous SD (white) and in which spontaneous SD occurred (colored). Each horizontal box represents the total number of experiments taken as 100%, and the number of rats is given in the middle of bars. (**E**) Drop of CBF after ischemia induction, depicted for each experiment involving pH-sensitive microelectrodes. (**F**) The relative amplitude of acidosis induced by ischemia alone, measured directly prior to the detection of SD, for each experiment involving pH-sensitive microelectrodes. Horizontal lines drawn in **E** and **F** indicate a theoretical level of threshold for the evolution of spontaneous SD.

The evaluation of spontaneous SDs delivered, perhaps, the most revealing findings of the present study. A single spontaneous SD event occurred within 2 min after ischemia induction in a total number of 15 out of 33 experiments. The distribution of these animals with respect to experimental methods and age groups is shown in Fig. 5D. Imaging identified that the spontaneous events entered the field of view at the frontal or lateral edges of the cranial window, being generated distant to SDs evoked with KCl application near the medial border of the cranial windows (Fig. 5). Overall, spontaneous SDs were observed more frequently in old animals (12 of 15 cases), but careful analysis of CBF and pH conditions around the time of spontaneous SD evolution suggested that a drop of CBF below 22–23% due to ischemia induction, or ischemia-induced acidosis greater than 0.2 pH units favored SD occurrence (Fig. 5E,F), rather than old age by itself. Also, pHe prior to ischemia induction was significantly more alkaline in cases when spontaneous SD occurred shortly after ischemia onset (electrophysiology: pH 7.28 ± 0.06 and 7.32 ± 0.05 vs. 7.21 ± 0.04, young with spont. SD and old with spont. SD vs. young with no spont. SD).

**Figure 6:**
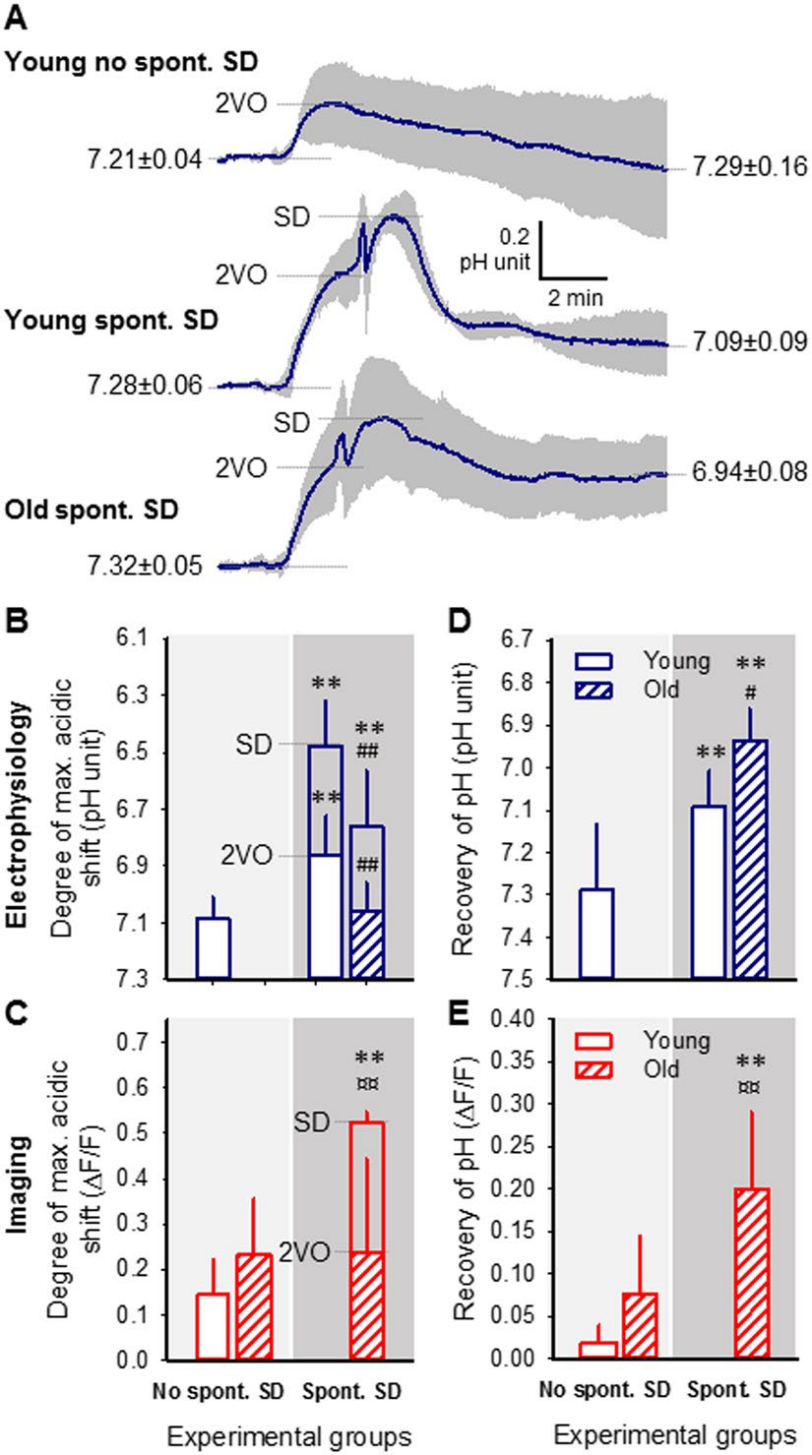
Tissue pH variations induced by ischemia and spontaneous spreading depolarization (SD). (**A**) Mean traces of tissue pH variation due to ischemia induction (2VO) and spontaneous SD are superimposed. Each trace is the average of individual ones taken from separate animals, and are presented as mean ± stdev (n = 6/8). In Panels B, C, D and E, the upper bar charts represent data obtained with pH-sensitive microelectrodes (blue), and the lower bar charts demonstrate data derived from Neutral Red (NR) fluorescence imaging (red). Light shaded background of the bar charts (left) labels experiments devoid of spontaneous SD, and dark shaded background in the bar charts (right) stands for experiments in which spontaneous SD occurred. (**B**,**C**) Amplitude of acidosis. (**D**,**E**) Tissue pH assessed 12 min after SD occurrence (i.e. tissue pH recovery). For the evaluation of statistical significance, a two-way analysis of variance (ANOVA) paradigm considering age and the occurrence of spontaneous SD as its factors was applied. Level of significance for the ANOVA was defined as *p < 0.05 and **p < 0.01. Significant differences between groups were determined by a Fisher post hoc test as follows: *p < 0.05 and **p < 0.01, vs. respective Young; ^#^p < 0.05 and ^##^p < 0.01, vs. respective with no spontaneous SD.

The consequences of spontaneous SDs were grave. The SD-related acidosis was superimposed on ischemia-induced acidosis (Fig. 6A) thereby transiently decreasing pHe from the ischemia-related value of 6.93 ± 0.09 to as low as pH 6.48 ± 0.16 in the young group, and from pH 7.06 ± 0.10 to 6.76 ± 0.20 in the old animals (Fig. 6B). The occurrence of spontaneous SD more than doubled NR fluorescence intensity with respect to that achieved by ischemia alone (Fig. 6C). Tissue pH in young animals measured over 10 min after spontaneous SD settled to a value more acidic than that produced by ischemia alone at a corresponding point of time (pH 7.09 ± 0.09 vs. 7.29 ± 0.16), which was further worsened by age, as pHe after spontaneous SD was maintained at an average of pH 6.94 ± 0.08 in the old animals (Fig. 6D,E).

## Discussion

Even though *in vivo*, live imaging of brain pHi with NR had been achieved previously^26, 27^, our approach presented here reaches further and is unique in that NR fluorescence indicative of pHi was supported by and correlated with the conventional pHe signal acquired with pH-sensitive microelectrodes.

Intracellular acidosis with SD, as indicated by NR fluorescence, must represent a sum of neuronal and astrocytic pH transients, assuming that both cell types incorporate the dye, and accepting that contribution of NR circulating in blood plasma is negligible^25^. During SD in the intact cortex, astrocytes display a considerable alkaline pHi shift of 0.28 pH units^28^. Much less is certain about distinct pHi variations of neurons, but a recent study has revealed that neurons undergo intracellular acidosis with SD onset, demonstrated by selectively loading these cells in neocortical brain slices with the pH-sensitive fluorescent dye BCECF^29^. Taken together, the SD-associated pHi signal of NR in the intact cortex is presumed to be dominated by neuronal acidosis versus astrocytic alkalosis.

We have found a good correlation between intra- and extracellular acidosis during SD (Fig. 2A). This stands in agreement with the finding that newly produced lactate is readily released from neurons and from astrocytes to the extracellular space by lactate-proton cotransport^30^, and with the suggestion that pHi and pHe decrease parallel at increasing lactate concentration in the ischemic tissue^31^. Tissue pH after the passage of the first SD remained elevated probably because it may take up to 45 min for lactate concentration to normalize following an SD event^32^.

Three elements of the pHe signature of SD did not appear on the pHi traces. The initial, brief acidic shift was most certainly an artefact created when the reference was subtracted from the raw pH signal, because the tips of the two electrodes could not possibly be located at an identical spot^10^. The subsequent alkaline shift was previously attributed to the efflux of HCO_3_^−^ from cellular compartments to the interstitium^10, 33^, or could be caused by a brief drop in lactate concentration^32^. Finally, the more gradual recovery of pHe with respect to pHi probably followed the rate of lactate recycling or extrusion to the blood stream^9^.

The current study has delivered three novel observations, with neurological consequences that are anticipated to be significant: (i) The SD-related acidosis is remarkably enlarged in the ischemic as compared with the intact cortex; (ii) SD-related acidosis evolving under penumbra-like conditions is additive to ischemia-induced acidosis.; (iii) In the aging brain, the recovery from SD-related acidosis is hampered, and tissue pH subsequent to SD remains more acidic with respect to the young cortex.

The intensification of SD-related acidosis under ischemia has been characterized here by the higher relative amplitude and longer duration of the acidic pH transients (Fig. 3). Previous observations suggest that tissue acidosis associated with SD in the intact cortex is primarily caused by lactate produced, because pHe was shown to decrease synchronously with the elevation of lactate^9, 10^. In support of the lactate-dependence of tissue acidosis, a linear correlation was established between increasing lactate levels and decreasing tissue pH in the ischemic brain^31, 34^. Based on the evidence listed here, we propose that the greater amplitude of SD-related acidosis in ischemic with respect to the intact cortex must be caused by the increased accumulation of lactate. Because lactate is generally the product of anaerobic metabolism, we speculate that lactate concentration, and thus the relative peak of acidosis associated with SD, depends on whether the tissue can initially utilize aerobic metabolic pathways in response to SD^7, 35^, or relies largely on anaerobic metabolism setting in before SD generation, because of ischemia^36^. The longer duration of SD-related acidosis and the underlying lactate production during ischemia, on the other hand, possibly correlate well with delayed repolarization^11, 22^, thus with the continuing demand for energy.

We have also demonstrated here that the SD-related acidosis is additive to ischemia-induced acidosis, especially with spontaneous SDs (Fig. 6). Our global forebrain ischemia model created conditions similar to the ischemic penumbra, in that CBF dropped below 40%, and remained between 20–40% until reperfusion was initiated^15, 37^. Prior to the detection of the propagating spontaneous SD, pHe ranged between pH 6.9–7.0 in the cortex, similar to pHi that was calculated for the penumbra region after middle cerebral artery occlusion (MCAO)^38^. Acidosis associated with SD here shifted pHe to as low as pH 6.48 in average, more typical of the ischemic core of focal insults. For comparison, focal ischemia upon onset produced tissue pH of 6.64 in the rabbit cortex^39^, and pH 6.53 at the peri-infarct rim after MCAO in rats^15^. Taken together, we propose that SDs that propagate over the ischemic penumbra transiently increase local acid load to cytotoxic levels typical of the ischemic core, which may be a potent pathophysiological mechanism to cause neurodegeneration. Furthermore, not only the amplitude but also the duration of acidosis was shown to jeopardize tissue integrity. As such, the prolongation of acid exposure was concluded to reduce the threshold of acid-induced cell death^40^. Taken that SDs occur in a recurrent fashion in ischemic zones^41, 42^, and that tissue pH remains acidic for at least 10 min after an SD event propagating under penumbra-like conditions (Fig. 6), SDs are suggested to prolong tissue acidosis, thereby also increasing the risk of neuronal injury.

The experimental protocol used might have led to the underestimation of the impact of SDs elicited after ischemia induction. SD is a known preconditioning stimulus, which induces ischemic tolerance^43^. From this point of view, the phase of baseline in our experiments can be perceived as a preconditioning period, which would attenuate the impact of subsequent ischemia. This may be reflected in the kinetics of the SD-related CBF response; for example, inverse neurovascular coupling was encountered frequently under ischemia in aged rats, when SDs were not triggered before ischemia induction^22^.

The spontaneous generation of SD was more frequently encountered in the old animals. Previously, we reported that spontaneous SDs emerged less frequently in the old brain than in the young in a rat model of focal ischemia, probably because longer-lasting depolarizations prevented the occurrence of subsequent SDs^20^. Here we show that SD occurs spontaneously when the perfusion deficit shortly after ischemia onset is severe (CBF drop to between 7–23%, albeit measured distant to the exact site of SD generation), which was encountered more frequently in old animals (Fig. 5E). These results corroborate the opinion that the aged nervous tissue is more susceptible to hypoxic or ischemic injury^23^, most certainly because its inherent sensitivity^44^ is challenged further by a deeper cerebral perfusion deficit after cerebral vessel obstruction. In turn, the supply-demand mismatch for energy substrates becomes aggravated, which makes the occurrence of SD more likely^12^. This initiates a vicious cycle more prominent in the aged brain, as SD deepens the metabolic crisis even further, and ultimately worsens tissue outcome^1, 11^. In summary, our results strongly indicate that in the aged brain the occurrence of SD is facilitated because the perfusion deficit is graver. Moreover, we propose that the aging brain may be at higher risk for acid-induced neurodegeneration, because tissue pH recovers slower and remains significantly more acidic after the passage of an SD than in the young brain (Fig. 6). Finally, the age-related metabolic pattern of SD evolution shown here is suggested to substantiate the accelerated conversion of ischemic penumbra into infarction with age, as was described earlier in patients^24^.

We found a strong positive correlation between the peak of acidosis and of hyperemia to SD in the young intact cerebral cortex (Fig. 4E). At close inspection, the imaging experiments revealed that the peak of acidosis preceded slightly the peak of hyperemia to SD. If these observations represent more than a coincidence, it would appear that the coupling during SD must have a metabovascular component, even though the prevalence of neurovascular over metabolic coupling with SD has been promoted^45^. Our data further indicate that if such a coupling exists, it is severely impaired in the aged brain (Fig. 4F).

Duration of acidosis and hyperemia, on the other hand, did not coincide in the intact cortex. We suspect that hyperemia disproportionately outlasts acidosis, possibly leading to a non-linear association. This would be consistent with the notion that the CBF response to SD creates luxury perfusion in the cortex that receives uninterrupted blood supply^45^. The mismatch may also imply that vasoactive mediators other than pH (e.g. arachidonic acid metabolites) are involved in regulating the CBF response to SD^45, 46^.

SD-related progression of neuronal injury in the ischemic penumbra was previously attributed to hypoperfusion in response to SD, known as inverse hemodynamic response or spreading ischemia^11^. The ruling hypoemic element of the CBF response to SD delays reporlarization and causes excitotoxic damage, ultimately leading to cell death^1, 11^. However, only a fraction of SDs propagating across the ischemic penumbra are coupled with spreading ischemia^22, 47^, and it has remained uncertain what mechanism might mediate neurodegeneration in case the hemodynamic response to SD is hyperemic. Here we present, that tissue pH drops remarkably with each SD propagating across the ischemic cortex, to which hyperemic CBF response is coupled. We propose that this acidic shift in tissue pH with recurrent SDs is additive and cytotoxic, consistent with the view that marked acid accumulation correlates with the extent of brain injury, and that tissue acidosis is a damaging component of cerebral ischemia^48^.

The long cumulative duration of ECoG depression associated with recurrent SDs has been suggested as an early marker of delayed ischemic brain damage^2, 49^. Based on our present results, tissue acidosis may be a sensitive indicator of potential tissue injury as well, and may be indirectly monitored in intensive care units by the use of lactate biosensors^50, 51^. We propose that the SD-associated, prominent, transient acidosis superimposed on ischemia-induced acidosis worsens tissue survival, thereby rendering SDs a causative, malignant event in cerebral ischemia. In contrast, the less pronounced pH changes with SDs in the non-ischemic cortex are suggested not to be directly harmful. Instead, SD-associated mild acidosis appears to be neuroprotective by suppressing the excitability of neurons and delaying the occurrence of subsequent SDs^17^.

## Materials and Methods

**Figure 7:**
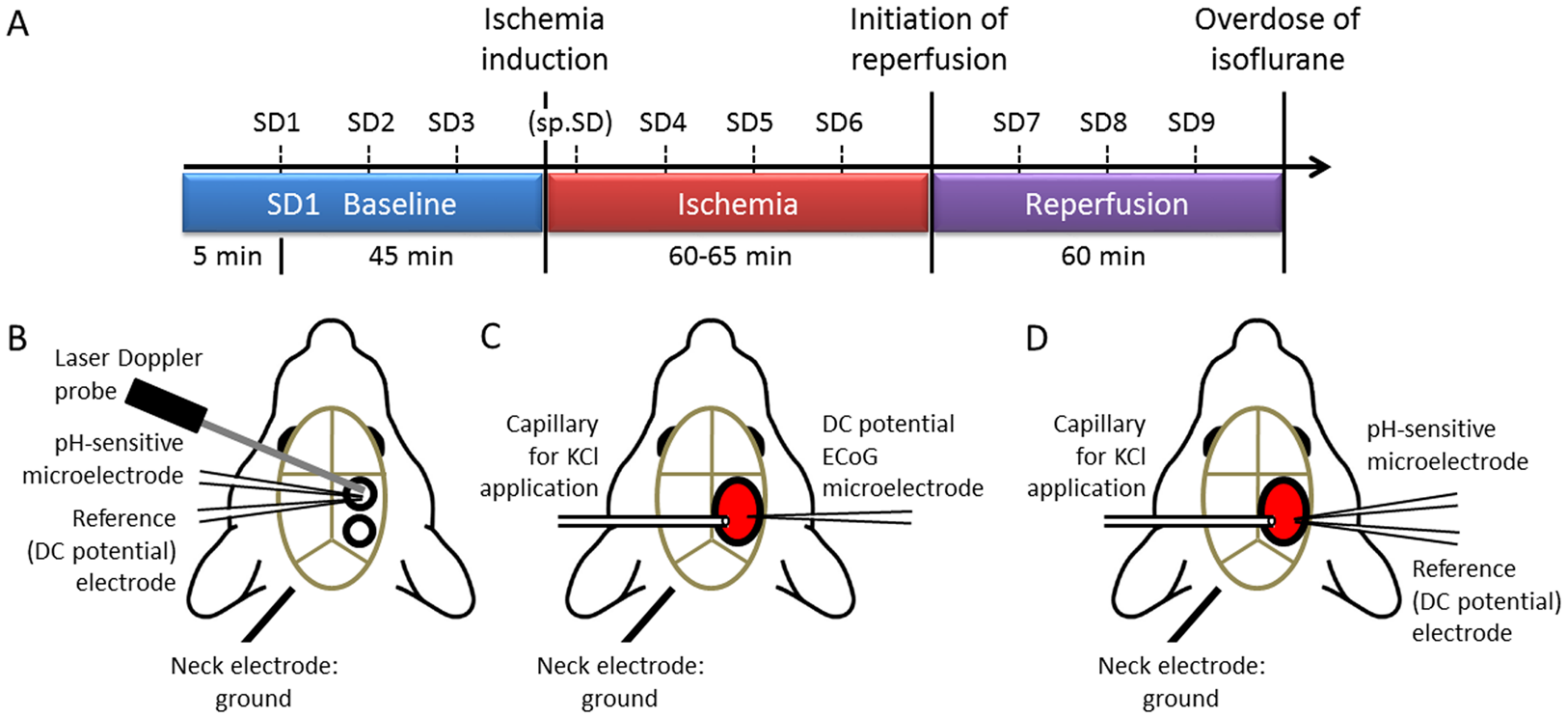
Experimental paradigm and models. (**A**) Schematic illustration of the experimental protocol. Numbered spreading depolarization events (e.g. SD1, SD2, … SD9) indicate SDs evoked by 1 M KCl application. Spontaneous SD (sp.SD) evolved in some but not all experiments, and is given in brackets due to its inconsistent occurrence. Ischemia and reperfusion was induced by the occlusion and latter release of both common carotid arteries. (**B–D**) Experimental preparation for the various data acquisition protocols used. (**B**) Conventional electrophysiological setting. Two open cranial windows were created (black circles) over the right parietal cortex. The frontal window served as recording site for data acquisition, and the caudal window was used for SD elicitation by the topical application of 1 M KCl. (**C**) Preparation for multimodal imaging (Neutral Red fluorescence and laser speckle contrast analysis). A closed cranial window was built over the parietal cortex and subsequently loaded with Neutral Red (black ellipse filled with red). The cranial window was equipped with a glass capillary to apply 1 μl of 1 M KCl topically to elicit SD (left), and a glass capillary microelectrode to acquire direct current (DC) potential and electrocorticogram (ECoG) (right). (**D**) Combination of multimodal imaging (shown in panel B) with conventional electrophysiology (shown in panel A).

The experimental procedures were approved by the National Food Chain Safety and Animal Health Directorate of Csongrád County, Hungary. The procedures conformed to the guidelines of the Scientific Committee of Animal Experimentation of the Hungarian Academy of Sciences (updated Law and Regulations on Animal Protection: 40/2013. (II. 14.) Gov. of Hungary), following the EU Directive 2010/63/EU on the protection of animals used for scientific purposes. Young adult (2 month-old, n = 20) and old (18–20 month-old, n = 18) male Sprague-Dawley rats were used. Anesthesia with isoflurane, general surgical procedures and monitoring of mean arterial blood pressure (MABP) were identical to those reported earlier^46, 47^. Animals were spontaneously breathing throughout the experimental protocol. Level of anesthesia was continuously controlled with the aid of MABP and ECoG displayed live as experiments were in progress. After a baseline period of 50 min, incomplete, global forebrain ischemia was induced by the bilateral occlusion of the common carotid arteries (2VO). An hour later, the carotid arteries were released to allow reperfusion of the forebrain for another hour. Experiments were terminated by the overdose of isoflurane. Three SDs were evoked with the topical application of 1 M KCl during each phase of the experiments (i.e. baseline, ischemia and reperfusion) at an inter-SD interval of 15 minutes (Fig. 7A). Samples for arterial blood gas analysis were withdrawn from the femoral artery before initiating the first SD, and under ischemia, before the release of the carotid arteries. For tissue pH monitoring, the animals were assigned to either implantation of a pH-sensitive microelectrode into the cortex (n = 17) (Fig. 7B), or a closed cranial window preparation over the parietal cortex for pH imaging (n = 20) (Fig. 7C). The combination of the two approaches was successfully achieved in a representative case (Fig. 7D).

### Application of pH-sensitive microelectrodes

Two craniotomies (5 mm apart) were prepared in the right parietal bone using a dental drill (Fig. 7B). The dura in each craniotomy was carefully dissected, and the craniotomies were regularly irrigated with artificial cerebrospinal fluid (aCSF; mM concentrations: 126.6 NaCl, 3 KCl, 1.5 CaCl_2_, 1.2 MgCl, 24.5 NaHCO_3_, 6.7 urea, 3.7 glucose bubbled with 95% O_2_ and 5% CO_2_ to achieve a constant pH of 7.4).

Ion-sensitive microelectrodes were prepared according to Voipio and Kaila (1993)^52^. In each experiment, a pH-sensitive microelectrode was lowered with a micromanipulator into the cortex, together with another glass capillary microelectrode (tip diameter = 20 μm) filled with saline to serve as reference (n = 17). The tips of the two electrodes were positioned as near as possible. The reference electrode acquired slow cortical or DC potential. An Ag/AgCl electrode was implanted under the skin of the animal’s neck to be used as common ground. Microelectrodes were connected to a custom-made dual-channel high input impedance electrometer (including AD549LH, Analog Devices, Norwood, MA, USA) via Ag/AgCl leads. The voltage signal recorded by the reference electrode was subtracted from that of the pH-sensitive microelectrode by a dedicated differential preamplifier (NL834) and forwarded to an associated four channel analog signal isolator amplifier (NL 820), which yielded potential variations related to changes in H^+^ ion concentration. The recorded signals were then forwarded to an analog-to-digital converter (MP 150, Biopac Systems, Inc) through filters (NL125) and conditioners (NL530). Electric signals via the electrodes were continuously acquired at a sampling frequency of 1 kHz, displayed live, and stored using a personal computer equipped with the software AcqKnowledge 4.2.0 (Biopac Systems Inc., USA). Extracellular pH (pHe) changes were expressed in mV to be translated into pH units offline, using least squares linear regression.

In order to assess changes in local CBF, a laser-Doppler needle probe (Probe 403 connected to PeriFlux 5000; Perimed AB, Sweden) was positioned at an angle adjacent to the intra-cortical microelectrode (n = 14) (Fig. 7A). The laser-Doppler flow (LDF) signal was digitized and displayed together with the DC potential and pH signals as described above (MP 150 and AcqKnowledge 4.2.0, Biopac Systems, Inc. USA). The completed preparations were enclosed in a Faraday cage.

The caudal craniotomoy was later used for SD elicitation by placing a 1 M KCl-soaked cotton ball on the exposed cortical surface. The cotton ball was removed and the cranial window rinsed with aCSF immediately after each successful SD elicitation.

### Neutral Red and cerebral blood flow imaging

A cranial window (4.5 × 4.5 mm) was created over the parietal cortex as described in detail earlier^53, 54^. In addition, a glass capillary microelectrode (outer tip diameter = 20 μm) was inserted into the cortex within the cranial window, near the lateral edge of the craniotomy, opposite to the site of later SD elicitation (Fig. 7C). The electrode was used to acquire DC potential with reference to an Ag/AgCl neck electrode, to confirm SD occurrence and propagation in the cortex. Signal amplification and filtering was carried out as reported earlier^47^.

Neutral Red (3-amino-m-dimethylamino-2-methylphenazine hydrochloride, NR, Sigma-Aldrich), whose fluorescence intensity increases with decreasing pH was dissolved in saline (35 mM), and administered i.p. (2 × 1 ml) 30–35 min prior to the start of imaging^25^.

For live imaging, the cortex was illuminated in stroboscopic mode (100 ms/s) with a high-power light emitting diode (LED) (530 nm peak wavelength; SLS-0304-A, Mightex Systems, Pleasanton, CA, USA) equipped with a bandpass filter (3RD 540–570 nm, Omega Optical Inc. Brattleboro, VT, USA). Emitted NR fluorescence passed through a 50 nm wide bandpass filter centered on 625 nm (XF3413–625QM50; Omega Optical Inc. Brattleboro, VT, USA), and was captured with a monochrome CCD camera (resolution: 1024 × 1024 pixel, Pantera 1M30, DALSA, Gröbenzell, Germany) attached to a stereomicroscope (MZ12.5, Leica Microsystems, Wetzlar, Germany). The stereomicroscope was equipped with a 1:1 binocular/video-tube beam splitter to allow the mounting of a second camera. Synchronous with NR images, green intrinsic optical signal (IOS) was captured at 100 ms exposure each second by the second CCD camera identical to that used for NR imaging, but equipped with no emission filter. In addition, both cameras recorded background at 1 frame per second frequency, to be used for the correction of raw images. A dedicated program written in LabVIEW environment synchronized the illumination and camera exposures. Finally, NR fluorescence was mathematically corrected offline, for the absorption of excitation and fluorescence wavelengths by hemoglobin^55^ and for the bleaching of NR.

NR imaging was combined with CBF imaging involving laser speckle contrast analysis (LASCA), as reported earlier^54^. Briefly, the cortex was illuminated with a laser diode (HL6545MG, Thorlabs Inc., New Jersey, USA; 120 mW; 660 nm emission wavelength) driven by a power supply (LDTC0520, Wavelength Electronics, Inc., Bozeman, USA) set to deliver a 160-mA current. The raw laser speckle images were captured by the second CCD camera. Laser illumination and camera exposure were synchronized (1 frame/second; 2 ms for illumination and 100 ms for exposure). Image processing was carried out in MATLAB (The MathWorks Inc., Natick, MA, USA). Briefly, CBF maps were computed from the corresponding raw images as reported earlier^56^, following the reduction of background noise by subtracting the local variance and local mean values of the background images from the corresponding raw speckle images.

### Data processing and analysis

SDs were divided into two major categories based on whether the event was triggered with KCl (evoked SDs), or occurred due to the induction of ischemia (spontaneous SDs). Evoked SDs were further subdivided into first SD (SD1), subsequent SDs during baseline, SDs under ischemia, and SDs during reperfusion. The impact of age was evaluated for both evoked and spontaneous SDs. Direct current potential, pH and CBF variations with SD were processed and evaluated as reported earlier^22^. Briefly, the amplitudes of depolarization, acidosis and hyperemia with SD were defined at the maximum deflection relative to baseline. The maximum rates of depolarization, acidosis and hyperemia were calculated as the respective slopes taken for a linear segment of the signal, fit to the inflexion point (i.e. most rapid change). The duration of the events was measured at half amplitude.

Local changes in NR fluorescence intensity and CBF with time were extracted by placing regions of interest (ROIs) of 19 × 19 pixel size (~70 × 70 μm) at selected sites devoid of any blood vessels visible in the images. CBF recordings obtained by LDF or LASCA were expressed relative to baseline by using the average CBF value of the first 240 s of baseline (100%) and the recorded biological zero obtained after terminating each experiment (0%) as reference points. Data are given as mean ± stdev. The software SPSS (IBM SPSS Statistics for Windows, Version 22.0, IBM Corp.) was used for statistical analysis. Distinct statistical methods are provided in detail in each Figure legend.
